# Risk Management Plans: reassessment of safety concerns based on Good Pharmacovigilance Practices Module V (Revision 2)—a company experience

**DOI:** 10.1186/s40780-022-00244-z

**Published:** 2022-05-05

**Authors:** Suzan Esslinger, Linda Quinn, Sami Sampat, Marijo Otero-Lobato, Wim Noël, Anja Geldhof, Nicole Herijgers, Sarah-Jane Reeder

**Affiliations:** 1 Medical Affairs, Cilag GmbH International, Gubelstrasse 34, 6300 Zug, Switzerland; 2 Global Medical Safety, Janssen Research & Development, LLC, 850 Ridgeview Drive, Horsham, PA 19044 USA; 3 Medical Affairs, Janssen Biologics BV, Einsteinweg 101, 2333 CB Leiden, The Netherlands; 4 EMEA Medical Affairs, Janssen Cilag NV, Turnhoutseweg 30, 2340 Beerse, Belgium; 5 EMEA Regulatory Affairs, Janssen Biologics BV, Archimedesweg 29, 2333 CM Leiden, The Netherlands; 6 Global Medical Safety, Janssen-Cilag Ltd., 50-100 Holmers Farm Way, High Wycombe, HP12 4DP Buckinghamshire, UK

**Keywords:** Risk Management Plan, Good Pharmacovigilance Practice, Module V, Safety concerns, Algorithm

## Abstract

**Introduction:**

In the European Union (EU), a Risk Management Plan (RMP) is submitted as part of the dossier for initial marketing authorization of a medicinal product or with an application involving a significant change to an existing marketing authorization. A comprehensive revision of the EU Guideline on Good Pharmacovigilance Practices (GVP) Module V—Risk Management Systems (Revision [Rev] 2), adopted in March 2017, provides a framework for developing more focused, actionable, and risk-proportionate RMPs. This paper describes the Janssen experience with the interpretation and application of GVP Module V (Rev 2) regarding the evaluation of safety concerns in an RMP.

**Methods:**

Janssen convened a cross-functional working group to promote consistent interpretation of the GVP Module V (Rev 2) guidance across therapeutic areas. The group created 3 algorithms to support implementation of the guidance related to removal or reclassification of safety concerns by product-specific RMP teams.

**Results:**

Following implementation of the GVP Module V (Rev 2) guidance, the algorithm-driven process led to a substantial decrease in the number of safety concerns for most products. With few exceptions, EU health authorities agreed with the proposed safety concern removals or reclassifications, resulting in RMPs that were focused on only those safety concerns that required further characterization or specific risk minimization.

**Conclusions:**

The algorithm-driven process allows for consistent interpretation and application of the GVP Module V (Rev 2) guidance, which enables product teams to develop an actionable RMP using a thoughtful, evaluative, science-based approach that considers all available evidence.

## Introduction

In the European Union (EU), a Risk Management Plan (RMP) must be submitted as part of the dossier for an initial marketing authorization application, with an application involving a significant change to an existing marketing authorization, or at the request of either the European Medicines Agency (EMA) or competent authority in a Member State [1–5]. The purpose of an RMP is to describe the risk management system for a medicinal product, with a focus on appropriate risk management planning throughout the product life cycle. To this end, an RMP documents the safety profile of a product, emphasizing (1) safety concerns requiring further evaluation and/or risk minimization, (2) pharmacovigilance (PV) activities to characterize the safety concerns, and (3) measures intended to prevent or minimize harm to patients [1–5]. Accordingly, an RMP is composed of 3 core elements: a Safety Specification, a PV Plan, and a Risk Minimization Plan [2, 3].

The RMP Safety Specification describes the safety profile of a product, i.e., what is known about the Important Identified Risks (IIRs), Important Potential Risks (IPRs), and Missing Information (MI) [2–4, 6]. An identified risk is defined as an undesirable clinical outcome for which there is adequate scientific evidence of a causal relationship with the medicinal product [3, 4]. A potential risk is defined as an undesirable clinical outcome for which there is scientific evidence to suspect the possibility of a causal relationship with the product, but the evidence is insufficient to confirm a causal relationship. Furthermore, the RMP is expressly focused on those identified and potential risks that are considered important, i.e., those risks that could impact the benefit-risk balance of the product or have implications for public health [3]. Missing Information refers to insufficient knowledge regarding the safety of a product for certain anticipated utilization (e.g., long-term use) or for use in specific patient populations. The IIRs, IPRs, and MI of a product are collectively known as safety concerns [2–4, 6].

The RMP PV Plan outlines activities aimed at further characterizing and quantifying the important risks of a product, identifying new important risks, and aggregating knowledge to address MI. In addition, the PV Plan includes specific activities designed to evaluate the effectiveness of additional Risk Minimization Measures (RMMs) captured in the Risk Minimization Plan. The PV Plan consists of 2 types of activities: (1) routine PV activities beyond adverse reaction reporting and signal detection and (2) additional PV activities (Table 1) [2–5].

**Table 1:** Pharmacovigilance activities for risk management planning

Activity
***Routine PV activities beyond adverse reaction reporting and signal detection***^**a**^
• TFUQs to obtain structured information on reported suspected adverse reactions of special interest • Other forms of routine PV activities - Enhanced passive surveillance system (high‐level description) - Observed versus expected analyses - Cumulative reviews of adverse events of interest
***Additional PV activities***
• Non‐clinical studies • Clinical trials or non‐interventional studies (e.g., long‐term follow up of patients from the clinical trial population or cohort study to provide additional characterization of the long‐term safety of a product, PASS) • Studies/surveys to evaluate the effectiveness of additional RMMs (in accordance with GVP Module XVI; see Table 2) [7]

*GVP* Good Pharmacovigilance Practices, *PASS* Postauthorization Safety Study, *PV* Pharmacovigilance, *RMMs* Risk Minimization Measures, *RMP* Risk Management Plan, *TFUQs*, Targeted Follow-up Questionnaires

^a^Only routine PV activities that go beyond adverse reaction reporting and signal detection should be included in the RMP

The Risk Minimization Plan of an RMP describes RMMs intended to minimize or mitigate important risks associated with a product, thereby maximizing patient safety by optimizing the benefit-risk profile. Similar to the PV Plan, the Risk Minimization Plan has 2 components: (1) routine RMMs, with a focus on recommendations for specific clinical measures to address important risks, and (2) additional RMMs considered essential for safe and effective product use (Table 2) [2–5, 7].

**Table 2:** Risk Minimization Measures for risk management planning

Activity
***Routine RMMs beyond standard clinical care***^**a**^
• Performing a test before the start of treatment • Monitoring of laboratory parameters during treatment • Monitoring for specific signs and symptoms • Adjusting the dose or stopping the treatment when adverse events are observed or laboratory parameters change • Performing a wash‐out procedure after treatment interruption • Providing contraception recommendations • Prohibiting the use of other medicines while taking the product • Treating or preventing the risk factors that may lead to an adverse event of the product • Recommending long‐term clinical follow‐up to identify delayed adverse events in early stages
***Additional RMMs***^**b**^
• DHPCs • Educational programs/materials for healthcare professionals and/or patients (e.g., administration guide, checklist for prescribing, patient card, patient educational leaflet) • Controlled access programs • Controlled distribution systems • Pregnancy prevention programs
***Evaluation of RMMs***
• RMMs included in the RMP should be re-evaluated periodically • Effectiveness of RMMs should be assessed in accordance with GVP Module XVI (see Table 1) [7]

*DHPCs* Direct Healthcare Professional Communications, *GVP* Good Pharmacovigilance Practices, *RMMs* Risk Minimization Measures, *RMP* Risk Management Plan

^a^Only routine RMMs that recommend specific activities/steps that go beyond what is already integrated in standard clinical care qualify for inclusion in the RMP (e.g., treatment protocols or clinical guidelines)

^b^Some additional RMMs (e.g., patient cards, controlled access programs, pregnancy prevention programs) might need to be retained for the lifetime of the product

### Brief history of EU pharmacovigilance

In 2005, the European Commission commenced a review of the EU PV system, with the main objective of reducing the significant health burden of adverse drug reactions [1, 5]. This led to major changes that strengthened PV processes and culminated in the adoption of Directive 2010/84/EU and Regulation (EU) No 1235/2010 in December 2010 [1, 8, 9]. The Commission Implementing Regulation No 520/2012, which provided guidance on the implementation and operationalization of the 2010 legislation, followed in June 2012 [10]. In parallel, the Guideline on Good Pharmacovigilance Practices (GVP) in the EU was developed by experts from the EMA and national competent authorities [5, 11]. The scope of the GVP includes both major PV processes and product- or population-specific considerations. The GVP module specific for risk management planning, Module V—Risk Management Systems, was first adopted in June 2012 [2]. This module represented a shift in focus from purely managing risks to understanding risks in the context of benefit. An initial revision to Module V, primarily to clarify terminology, became effective in April 2014 (GVP Module V Revision [Rev] 1) [12].

A second revision of GVP Module V (i.e., Rev 2), which was significantly more comprehensive and far-reaching than Rev 1, was adopted in March 2017 following extensive feedback from stakeholders involved with or impacted by the risk management process [3, 4]. This heralded a paradigm shift in the approach to risk management planning.

Fundamentally, GVP Module V (Rev 2) introduced 3 major changes: (1) additional clarification of the focus of the RMP with respect to the safety concerns to be included; (2) a heightened focus on the dynamic nature of the RMP, including considerations for the reclassification and removal of safety concerns throughout the product life cycle; and (3) updated requirements to support the development of risk-proportionate RMPs based upon the type of initial marketing authorization application (e.g., generic products, fixed combination products, and biosimilar products) [3, 4, 13, 14]. The remainder of this paper addresses the impact of changes (1) and (2) and the practicalities of their implementation, as these provided the key challenges for Industry within this revised GVP guidance.

### Change 1: focused Risk Management Plan safety concerns

Although important risks continue to be defined by their proven or potential impact on the benefit-risk balance of a product, GVP Module V (Rev 2) increased emphasis on their clinical impact and targeted risk management planning (Table 3). Previously, important risks were defined as untoward occurrences for which there was evidence of, or some basis for suspicion of, an association with the product [2, 12]. GVP Module V (Rev 2) requires important risks to be oriented toward a concrete undesirable clinical outcome. Furthermore, only those important risks requiring further evaluation and characterization and/or those requiring specific RMMs should be included in the RMP. Finally, GVP Module V (Rev 2) clarified that MI should be limited to gaps in knowledge about the safety of a product when used within the approved indication(s) and for which there is a scientific basis to suspect a different safety profile from that characterized so far [3, 4, 13, 14].

**Table 3:** Summary of changes in the Guideline on Good Pharmacovigilance Practices Module V (Revision 2) (including Guidance on the format of the Risk Management Plan in the European Union—in integrated format) [3, 4, 13, 14]

Safety Concern	Summary
**IIRs**	***Impact on RMP Planning***
	The RMP should address only those IIRs which are undesirable clinical outcomes. An IIR to be included in the RMP would usually require: • Further evaluation/characterization as part of the PV Plan • RMMs: such as product information advising on specific clinical actions to be taken to minimize the risk or additional RMMs)
	***Considerations for Removal***
	An IIR may be removed from the RMP where: • Fully characterized, with no outstanding additional PV activities • Appropriately managed, with no ongoing additional RMMs and where any RMMs recommending specific clinical measures have become fully integrated into standard clinical practice
**IPRs**	***Impact on RMP Planning***
	The RMP should address only those IPRs which are undesirable clinical outcomes. An undesirable clinical outcome associated with off-label use or an area of MI may be included as an IPR if deemed important and supported by a scientific rationale An IPR to be included in the RMP would usually require: • Further evaluation/characterization as part of the PV Plan
	***Considerations for Removal***
	An IPR may be reclassified or removed from the RMP where: • Scientific and clinical data strengthen evidence of a causal association with the product [reclassification to IIR] • Accumulating scientific and clinical data do not support a causal association with the product • There is no reasonable expectation that any PV activity could further characterize the risk)
**MI**	***Impact on RMP Planning***
	Areas of MI included in the RMP should: • Be within the approved indication • Focus on areas where there are gaps in knowledge about the safety for certain anticipated utilization/patient populations • Be based upon a scientific rationale that anticipates the areas of MI might differ from the known safety profile MI in the RMP would usually require^a^: • Further evaluation/characterization as part of the PV Plan
	***Considerations for Removal***
	An area of MI may be removed from the RMP where: • Adequate safety data are available with respect to the area of MI • There is no reasonable expectation that any PV activities could further characterize the safety profile with respect to the areas of MI

*B-R* Benefit-Risk, *EU* European Union, *GVP* Good Pharmacovigilance Practices, *IIRs* Important Identified Risks, *IPRs* Important Potential Risks, *MI* Missing Information, *PV* Pharmacovigilance, *RMMs* Risk Minimization Measures, *RMP* Risk Management Plan

^a^Not specifically described in GVP Module V (rev 2), but based on information provided during the EMA information day

The result of this significant change in focus is an RMP that is more thoughtfully considered, succinct, actionable, and evidence driven. This is in contrast to previous RMP guidance, which often resulted in extensive lists of safety concerns, many of which did not require active risk management [2, 3, 12–14].

### Change 2: considerations for reclassification and removal of safety concerns

Although the RMP was conceived of as a planning document, its precise role within the PV landscape and how it was to evolve over time were uncertain. The introduction of GVP Module V (Rev 2) provided much-needed clarification as to the dynamic nature of risk management planning; namely, it is a continuous process that occurs throughout the life cycle of a product that is initiated in the preauthorization phase, evaluated during the marketing authorization application, and continued throughout the postauthorization phase [2–4, 13, 14]. As a product matures, the RMP is expected to change as knowledge regarding the benefit-risk profile of the product is gained [3, 4, 15]. Ultimately, this will result in modifications to the safety concerns included in the RMP over time. Unlike its predecessors, GVP Module V (Rev 2) provides considerations for the reclassification and removal of safety concerns within an RMP, based upon the need for and the feasibility of their further evaluation and/or need for specific RMMs (Table 3) [3, 4]. How this guidance is implemented is largely at the discretion of marketing authorization applicants and marketing authorization holders.

GVP Module V (Rev 2), therefore, represents an important shift in the approach to risk management planning in the EU [3, 4]. With its release, the EMA has provided guidance that allows marketing authorization applicants and marketing authorization holders to propose safety concerns that are based on scientific evidence, along with associated PV activities and RMMs that are feasible and proportionate to the benefits and risks of the product. This paper describes how Janssen has applied the GVP Module V (Rev 2) guidance to develop product-independent algorithms that drive consistent, comprehensive re-evaluation of RMPs, with particular attention to the reclassification and removal of safety concerns.

## Methods

### Development of algorithms (2017)

Shortly after GVP Module V (Rev 2) became effective in March 2017, Janssen convened a cross-functional working group, with members from Global Medical Safety Medical Affairs, Regulatory Affairs, and Clinical Research & Development, to assess the impact of the guidance and to develop a consistent approach for its implementation across therapeutic areas and throughout product life cycles. Individuals from Medical Affairs and Global Medical Safety were assigned to attend the EMA Information Day on Risk Management Planning (event #17,594, EMA, London, United Kingdom, December 19, 2017) where additional clarity around the requirements and management of each type of safety concern was provided; in particular with reference to MI and the expectation for this to be addressed with appropriate additional PV activities.

As an outcome of its impact assessment and considering the information provided during the EMA Information Day on Risk Management Planning, the algorithm-developing working group drafted 3 pilot algorithms—1 for each type of safety concern (i.e., IIR, IPR, and MI)—to facilitate the evaluation of safety concerns in Janssen product RMPs against the new guidance. Each algorithm included questions based on GVP Module V (Rev 2) to determine whether safety concerns should be reclassified, removed, or retained. Depending upon the answer to each question, the algorithm ultimately guided the product-specific RMP teams to a final decision, based on sound medical judgement after considering the totality of available evidence.

The 3 pilot algorithms were tested during planned RMP updates for 2 Janssen products that had longstanding (> 10 years) market approval, were within the same drug class, and had similar safety concerns [16]. After the algorithms were applied, each RMP was updated and reviewed by the respective product-specific RMP team. Comparison of the final RMP team decisions regarding reclassification, removal, and retention of safety concerns between the 2 products showed good alignment, underscoring the validity of the approach.

**Fig. 1:**
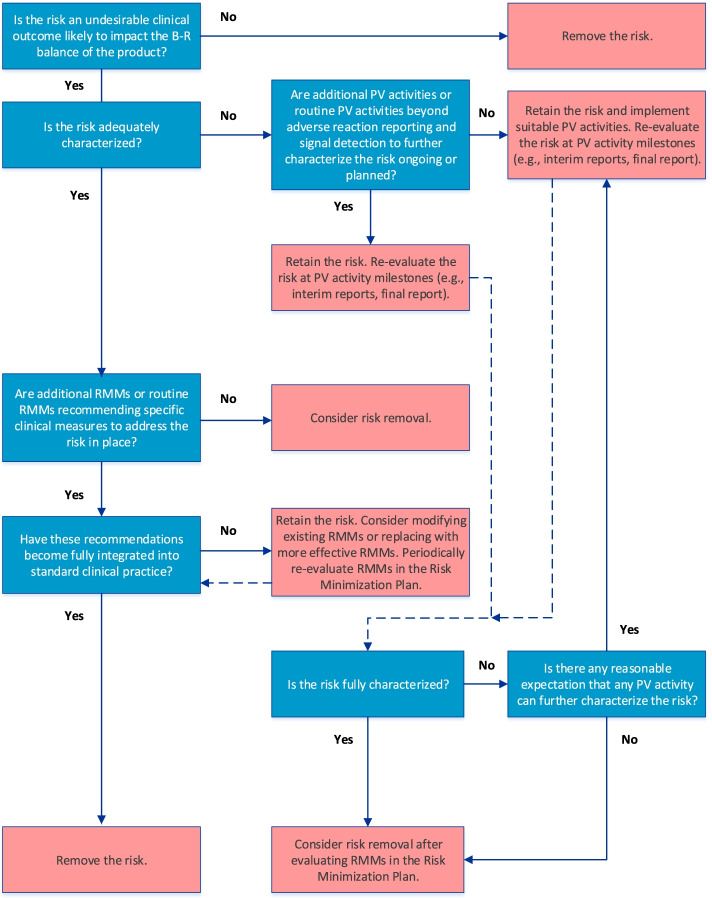
Algorithm for evaluation of Important Identified Risks. Dark gray boxes detail the guiding questions of the algorithm; light gray boxes detail the decisions (dependent upon the answer to the question). B-R, benefit-risk; PV, pharmacovigilance; RMMs, Risk Minimization Measures

**Fig. 2:**
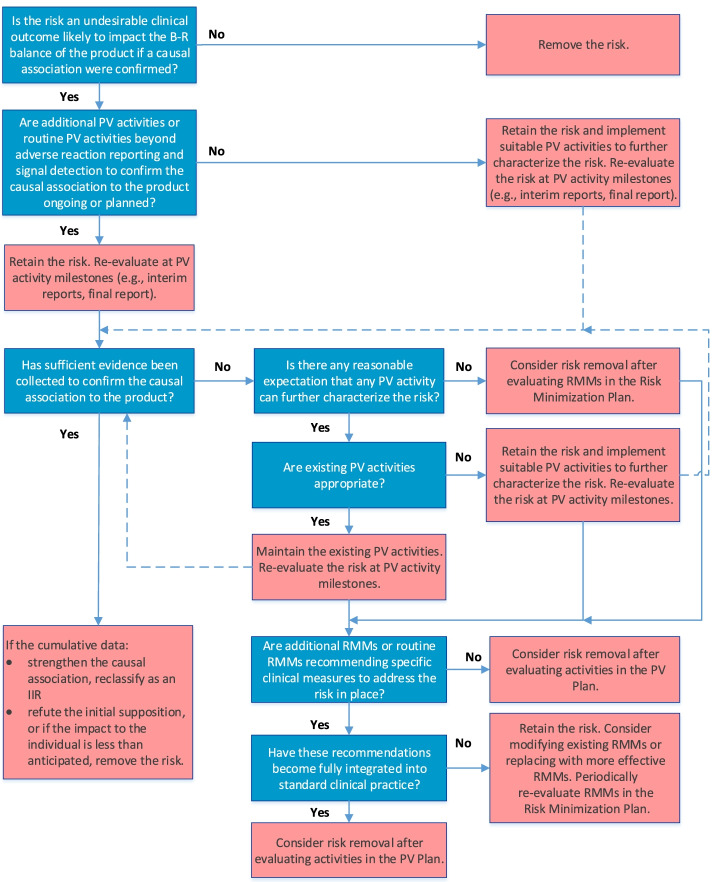
Algorithm for evaluation of Important Potential Risks. Dark gray boxes detail the guiding questions of the algorithm; light gray boxes detail the decisions (dependent upon the answer to the question). B-R, benefit-risk; IIR, Important Identified Risk; PV, pharmacovigilance; RMMs, Risk Minimization Measures

**Fig. 3:**
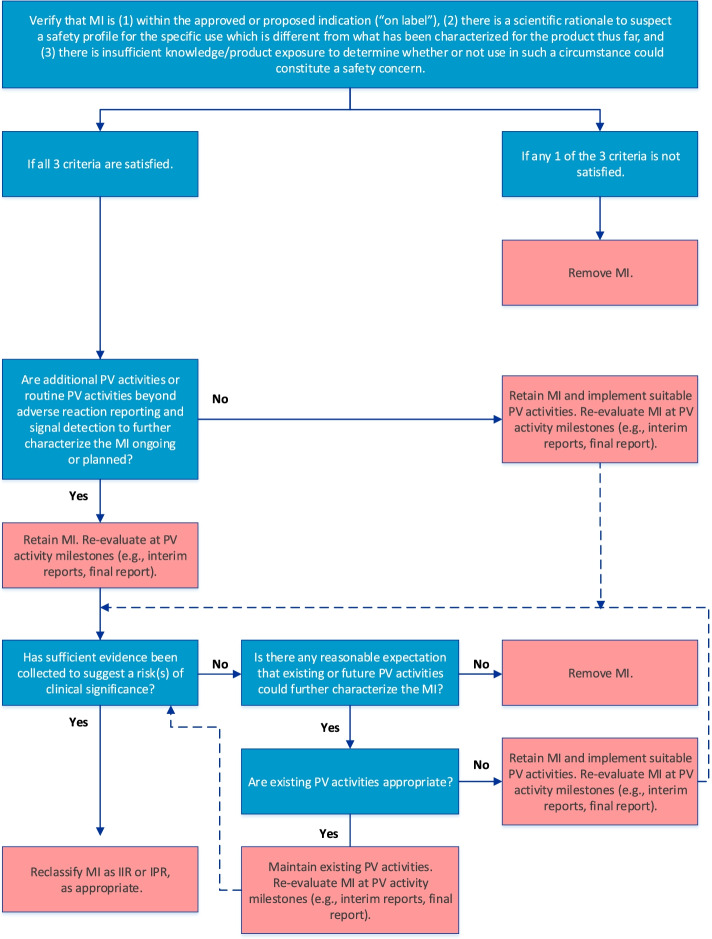
Algorithm for evaluation of Missing Information. Dark gray boxes detail the guiding questions of the algorithm; light gray boxes detail the decisions (dependent upon the answer to the question). B-R, benefit-risk; IIR, Important Identified Risk; IPR, Important Potential Risk; MI, Missing Information; PV, pharmacovigilance

Feedback from the product-specific RMP teams who applied the pilot algorithms and from internal and external stakeholders (e.g., EMA), combined with an increased familiarity with GVP Module V (Rev 2), informed the creation of the final algorithms (Figs. 1, 2, and 3; approved in February 2019) that are currently in use at Janssen.

### Algorithm for Important Identified Risks

The final algorithm for evaluating IIRs is depicted in Fig. 1. GVP Module V (Rev 2) defines IIRs as undesirable clinical outcomes that are likely to impact the benefit-risk balance of a product and for which there is sufficient evidence that they are caused by the product [3, 4]. In keeping with the overall objective of an actionable RMP, only IIRs that require further characterization (e.g., to determine frequency, severity, seriousness, or outcome of the risk under normal conditions of use or to identify populations particularly at risk) and/or specific RMMs should be included in the RMP.

The first step in the algorithm establishes whether the IIR fulfills the GVP Module V (Rev 2) definition. If it does, the next step in the algorithm, which considers whether the IIR is adequately characterized, is applied. Such characterization may be achieved through routine PV activities beyond adverse reaction reporting and signal detection and/or additional PV activities (Table 1). If the IIR requires further characterization, and if activities to address this are already included in the PV Plan, the IIR should be retained and re-evaluated at key PV activity milestones (e.g., interim or final study reports). Although not specified in the algorithm, if existing PV activities are deemed inadequate at the time of evaluation, they should be modified or replaced. If no activities to investigate the IIR are ongoing or planned despite the need for further characterization, suitable PV activities should be implemented.

After the need for further characterization of the IIR is determined, the next step in the algorithm considers whether routine RMMs recommending specific clinical measures to address the risk and/or additional RMMs (Table 2) are in place. If the IIR is considered fully characterized, it should be retained in the RMP only if RMMs in the Risk Minimization Plan have not yet been fully integrated into standard clinical practice; otherwise, the IIR should be removed from the RMP. The RMMs should be re-evaluated periodically for effectiveness and the need for continued implementation, as suggested in the GVP Module V (Rev 2). If necessary, the RMMs should be modified or replaced with more effective measures.

### Algorithm for Important Potential Rrisks

The final algorithm for evaluating IPRs is depicted in Fig. 2. GVP Module V (Rev 2) defines IPRs as undesirable clinical outcomes with a suspected causal association to the product, which, if confirmed, would likely impact the benefit-risk balance of the product [3, 4]. Accordingly, the first question in the IPR algorithm establishes the likelihood of the potential risk to impact the benefit-risk balance of the product if a causal association to the product were confirmed; only those potential risks for which the answer is “yes” are considered IPRs and should be retained in the RMP.

Because IPRs usually require further evaluation as part of the PV Plan, the algorithm next considers whether routine PV activities beyond adverse reaction reporting and signal detection and/or additional PV activities to investigate the IPR are ongoing or planned. If they are not, suitable PV activities to characterize the risk further should be implemented. If PV activities are already included in the PV Plan, the IPR should be re-evaluated at key PV activity milestones to determine whether sufficient evidence has been obtained to confirm or refute causality. If existing PV activities are deemed inadequate at the time of evaluation, they should be modified or replaced. Moreover, if cumulative data confirm a causal relationship, the IPR should be reclassified as an IIR; conversely, if cumulative data disprove a causal relationship, or if the data suggest that the impact to the benefit-risk balance is less than anticipated (i.e., the risk is not “important”), the risk should be removed from the RMP.

If it is determined that no PV activity could further characterize the IPR, the IPR may be removed from the RMP if no measures to minimize the risk are required in the Risk Minimization Plan.

If routine RMMs recommending specific clinical measures to address the risk and/or additional RMMs (Table 2) are in place, they should be evaluated to determine whether they have become integrated into standard clinical practice or require modification or replacement with more effective strategies. As for IIRs, the RMMs should be evaluated periodically for effectiveness and the need for continued implementation.

### Algorithm for Missing Information

The final algorithm for evaluating MI is depicted in Fig. 3. GVP Module V (Rev 2) defines MI as gaps in knowledge about the safety of a product for certain anticipated utilization or for use in particular patient populations for which there is insufficient knowledge to determine whether the safety profile differs from that characterized so far [3, 4]. For the purposes of risk management planning, MI should be included as a safety concern only if the following 3 criteria are satisfied, as stipulated in the first step of the algorithm: (1) the MI is within the approved or proposed indication as per the product label, (2) there is a scientific rationale to suspect a different safety profile, and (3) there is insufficient product knowledge or exposure to determine whether use of the product in a particular setting or patient population might be associated with risks of clinical significance.

The RMP should include a strategy for obtaining information on the benefit-risk balance where areas of MI exist, which may be achieved through routine PV activities beyond adverse reaction reporting and signal detection and/or additional PV activities. Therefore, the next step in the algorithm confirms whether such activities are already included in the PV Plan. If they are not, suitable PV activities should be implemented; otherwise, the MI should be re-evaluated at key PV activity milestones to determine whether sufficient evidence has been obtained to suggest an important risk, in which case reclassification as an IIR or IPR may be warranted. If the cumulative evidence suggests that the safety profile is not different, the MI should be removed from the RMP. Moreover, if, after a reasonable amount of time on the market, it is determined that no existing or future PV activities could further characterize the safety profile of the product with respect to the MI, the MI should be removed from the RMP.

### Roll-out and application of the algorithm-driven process across the company (2018–2020)

**Fig. 4:**
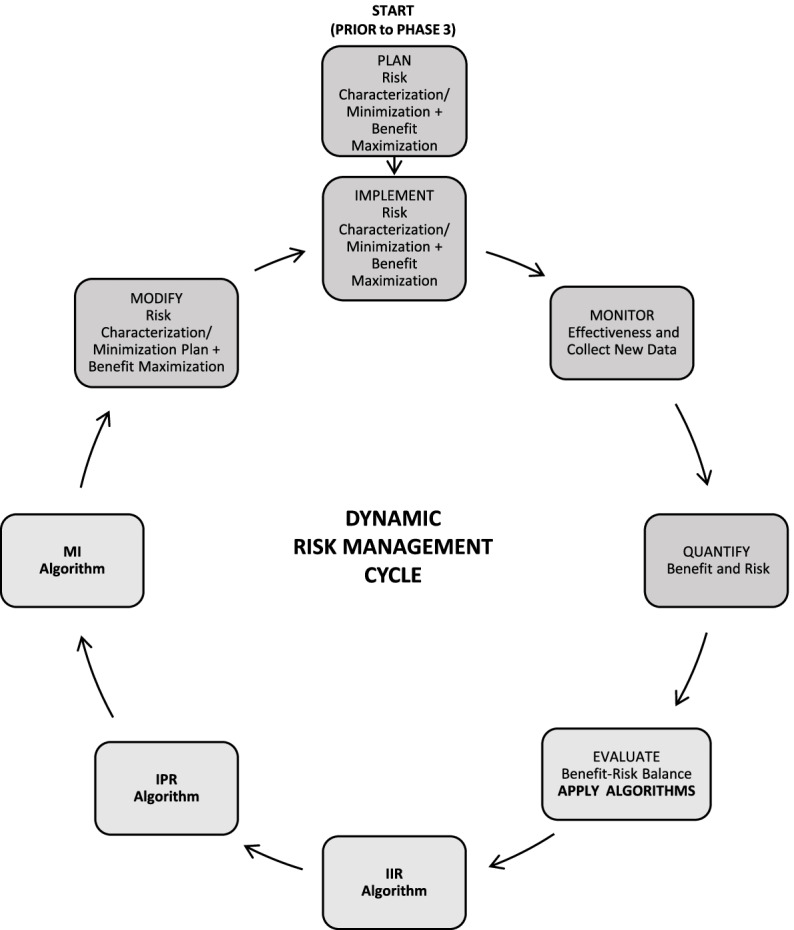
Dynamic cycle of risk management planning, Janssen algorithms inserted. Figure modified from [2, 12]. *Note:* RMPs are to be reviewed and updated throughout the life cycle of the product [2, 3, 12]. RMP updates may be triggered by the following [2, 3, 12, 15]: • HA or EMA request; • an application involving a change to the existing marketing authorization (e.g., new or significant indication change, new dosage form, new route of administration, or new manufacturing process of a biotechnologically derived product); • new data (including PSUR data) leading to a change in the list of safety concerns or addition of a new or a significant change to an existing additional PV activity or risk minimization measure, including the removal of a PV activity or risk minimization measure; • renewal of the marketing authorization. B-R, benefit-risk; HA, health authority; IIR, Important Identified Risk; IPR, Important Potential Risk; MI, Missing Information; PRAC, Pharmacovigilance Risk Assessment Committee; PSURs, Periodic Safety Update Reports; PV, pharmacovigilance; RMPs, Risk Management Plans

**Fig. 5:**
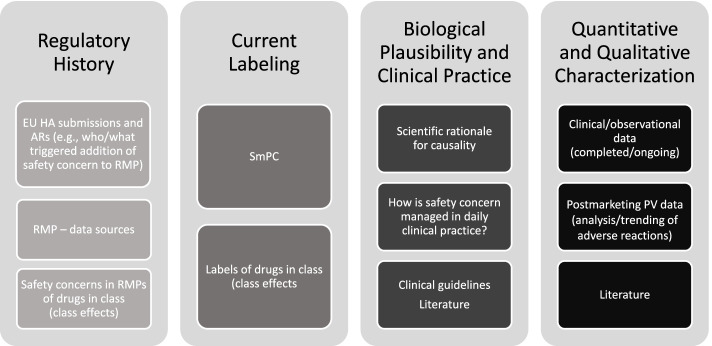
Information to consider when evaluating safety concerns for reclassification or removal from the RMP. AR, Assessment Report; EU HA, European health authorities; PV, pharmacovigilance; RMP, Risk Management Plan; SmPC, Summary of Product Characteristics

The Janssen working group prepared an informational pack consisting of the final algorithms (Figs. 1, 2, and 3) with operational footnotes (Tables 1 and 2), along with a training slide deck and high-level coaching tools (Figs. 4 and 5) to provide additional guidance to product-specific RMP teams [3, 4]. Figure 4 depicts the dynamic nature of the RMP process and includes specific points in the product life cycle at which the Safety Specification should be re-assessed and where the Janssen algorithms fit within the process. Figure 5 lists the spectrum of sources (regulatory history, clinical or observational trial data, product information, trending analyses from the global safety database, postmarketing safety data, results from additional PV activities, and the scientific literature) that product-specific RMP teams should consider when applying the algorithms.

Prior to re-evaluating RMPs, product-specific RMP teams are advised to review relevant EU health authority (HA) assessment reports and requests related not only to the product RMP under evaluation, but also to RMPs for other Janssen products and non-Janssen products in the same class. For each safety concern proposed for removal or reclassification utilizing the final algorithms, product-specific RMP teams are required to provide adequate justification along with appropriate reference to the safety data, EU HA assessment report, and EU HA request (including reference to the relevant procedure).

## Results

### Quantitative and qualitative evaluation of Janssen Risk Management Plans

To assess the performance of the algorithm-driven process for RMP re-evaluation, information from RMP Annex 8 (Summary of Changes to the Risk Management Plan Over Time) was analyzed to determine the number of safety concerns in each product-specific RMP before application of the algorithms and the number of safety concerns removed or reclassified following EU HA assessment and approval. If more granular information was needed, additional details were obtained from the respective product-specific RMP team.

**Fig. 6:**
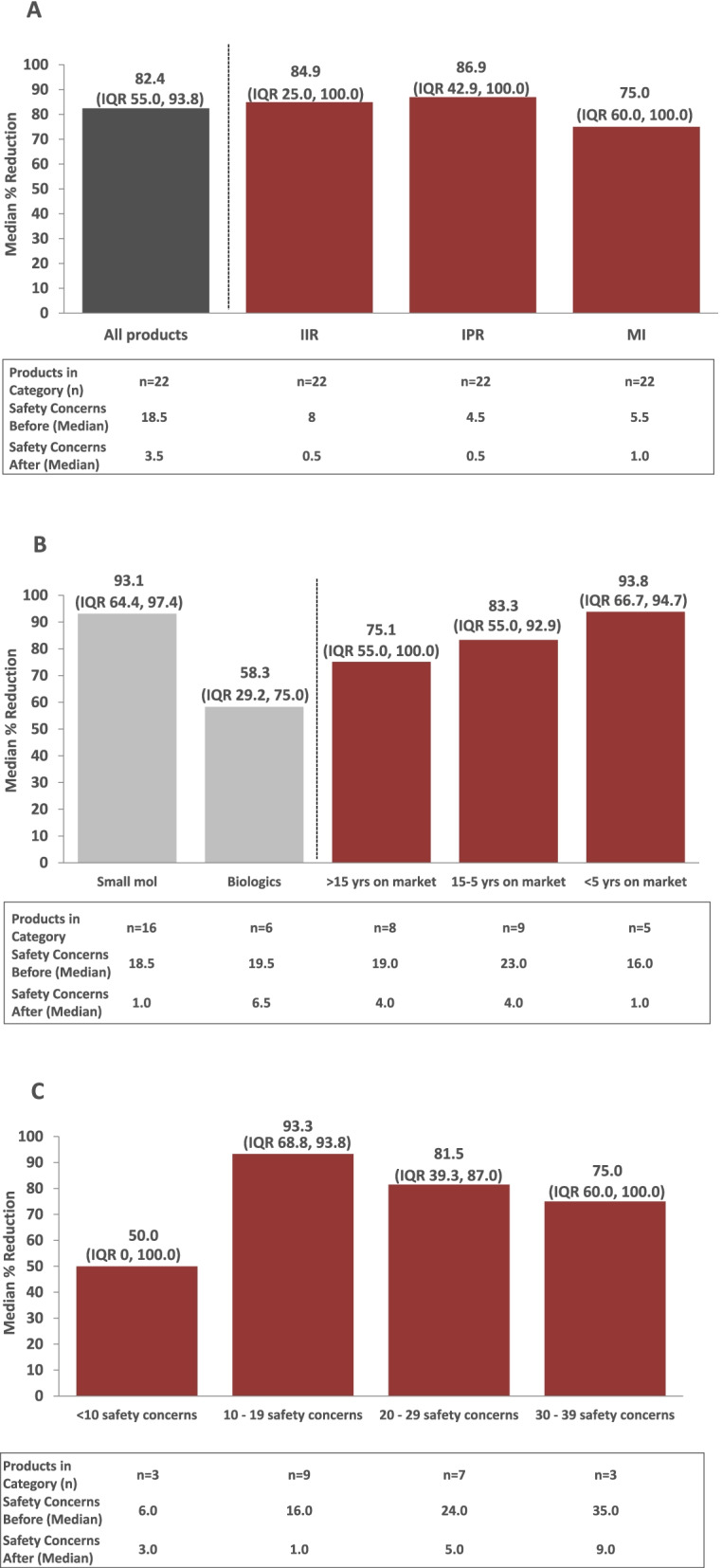
Reduction of safety concerns by type of safety concern (**A**), product type and market launch (**B**), and number of safety concerns (**C**). **A** The dark grey bar on the left shows the median % reduction of safety concerns across all 3 categories. The 3 red bars on the right are subsets of the total shown on the left, providing the median % reduction by category of safety concerns (IIR vs. IPR vs. MI). For products that did not include a specific category of safety concerns, the % reduction for this category was imputed as 0. **B** The 2 lighter gray bars on the left and the 3 red bars on the right are subsets of the total shown in dark grey in Fig. 6A on the left. The 2 lighter gray bars show the median % reduction of safety concerns by product type (small molecule vs. biologic); the 3 red bars show the median % reduction of safety concerns by time on the market. **C** The red bars are subsets of the total shown in grey in Fig. 6A on the left, providing the median % reduction of safety by the number of safety concerns in the RMP before applying the algorithms. IIR, Important Identified Risk; IPR, Important Potential Risk; MI, Missing Information; n, number of RMPs analyzed; RMPs, Risk Management Plans; Small mol, small molecule; yrs, years

Between March 2018 and March 2020, a total of 26 RMPs pertaining to 22 Janssen products were re-evaluated via the algorithm-driven process and subsequently submitted to EU HAs for assessment. For 4 out of 22 of these products, RMPs were re-evaluated in 2 separate procedures after new data became available. Results for the different RMPs for these 4 products were integrated per product for the purpose of this analysis (i.e., a total of 22 RMPs were included in the final analysis). Prior to application of the algorithms, the 22 RMPs evaluated included a median of 18.5 (interquartile range [IQR] 15.0, 24.0) safety concerns. After application of the algorithms and following final assessment by EU HAs, the median number of safety concerns per RMP dropped to 3.5 (IQR 1.0, 9.0). As shown in Fig. 6A, the median % reduction in safety concerns was 82.4% (IQR 55.0%, 93.8%). Median % reductions were similar across all 3 types of safety concerns (IIR: 84.9% [IQR 25.0%, 100%]; IPR: 86.9% [IQR 42.9%, 100%]; MI: 75.0% [IQR 60.0%, 100%]).

Eight of the 22 RMPs evaluated involved products that were marketed for more than 15 years (Fig. 6B). Nine RMPs involved products that that were on the market between 5 and 15 years. The remainder of RMPs (*n* = 5) involved products that were launched within the preceding 5 years. Median % reductions in safety concerns were generally similar across RMPs, irrespective of length of time since the product received marketing approval (75.1% to 93.8%; Fig. 6B). When the median % reduction in safety concerns among RMPs was analyzed by product type (small molecule versus biologic; *n* = 16 vs. *n* = 6, respectively; Fig. 6B), reductions tended to be greater among RMPs for small molecules (93.1% [IQR 64.4%, 97.4%]) compared with biologics (58.3% [IQR 29.2%, 75.0%]); this was presumably driven by 2 RMPs for biologics that included only a few safety concerns.

An analysis of the median % reduction in safety concerns by the number of safety concerns included in the RMP prior to application of the algorithms supported this conclusion. RMPs with 10 to 19, 20 to 29, and 30 to 39 safety concerns prior to application of the algorithms had greater median % reductions in safety concerns (93.3% [IQR 68.8%, 93.8%]; 81.5% [IQR 39.3, 87.0%]; and 75% [IQR 60.0%, 100%], respectively) compared with those with < 10 safety concerns (50%; [IQR 0, 100]), indicating that the number of safety concerns at the outset of the exercise may have played a role (Fig. 6C). However, the number of products with < 10 safety concerns was small (*n* = 3).

The reasons driving the decision for removal or reclassification varied by type of safety concern, as shown in Table 4. For important risks (IIRs and IPRs), common reasons for removal were that the risk was fully characterized, with no additional PV activities ongoing or planned, or that further characterization was not anticipated, e.g., due to the rarity of the event. Other frequent reasons for removing important risks were that no additional RMMs were in place or that, if they were in place, the additional RMMs were no longer required because guidance for management of the risk had been fully integrated into clinical practice. A common reason for the removal of safety concerns classified as MI was that they no longer met the revised GVP Module V (Rev 2) definition of MI; for example, the MI was not within the approved indication or there was no scientific rationale to suspect a different safety profile.

**Table 4:** Rationales for successful reclassification or removal of a safety concern

Safety Concern	Rationales and Examples
**IIRs or IPRs**	***Risk Characterization***
	• Risk well-characterized; additional information not expected: - Risk of “Severe skin reactions” removed for combination product composed of active substances well-characterized in the meantime • No reasonable expectation that additional PV activities could further characterize the risk: - Risk of “Guillain-Barré syndrome” due to rarity of event • Causal association not supported [IPR]: - Risks of “Congestive Heart Failure” and “Anemia” removed as causal relationship not supported by accumulated scientific and clinical data
	***Risk Management/Minimization***
	• Risk managed by routine RMMs; additional RMMs not required: - Risks of “Hyperglycemia” and “Urinary Tract Infection” removed as fully characterized and appropriately managed through product labels • Management of risk is fully integrated into routine clinical practice: - Risks with published management guidelines removed, e.g. “Lipid Abnormalities” • Risk is a known effect of mature product class: - Risk of “Hypertension” removed, as known to be associated with product class
	***Risk Not Compatible with Regulatory Definitions***
	• Risk not an undesirable clinical outcome: - Risk of “Medication error” removed; clinical consequences of medication errors captured under other IIRs - “Exposure during pregnancy” with no evidence of maternal/infant sequelae; reclassified to MI
	***Other***^**a**^
	• Subsuming of risks with the same underlying scientific concept: - Risk of “Opportunistic infections” subsumed under “Serious infections”
**MI**	***MI Characterization***
	• Adequate safety data available with respect to area of MI: - “Long-term safety” removed as MI once sufficient data available to confirm safety profile with long-term use • No reasonable expectation that additional PV activities could further characterize safety profile within area of MI: - “Use in the elderly” removed as MI where there remains insufficient exposure as condition prevalent in the young
	***MI Not Compatible with Regulatory Definitions***
	• MI not within approved indication: - “Use in pediatric patients” removed for products not authorized in this population - “Use in nursing mothers” removed for products contraindicated in this population
	• No scientific rationale for different safety profile: - “Use in patients with severe renal/hepatic impairment” removed for products with minimal renal clearance/hepatic metabolism
	***Other***^**a**^
	• Concept already covered by IIR/IPR: - “Use in patients with a history of malignancy” removed if malignancy was already listed as an IIR/IPR

*B-R* Benefit-Risk, *IIRs* Important Identified Risks, *IPRs* Important Potential Risks, *MI* Missing Information, *PV* Pharmacovigilance, *RMMs* Risk Minimization Measures

^a^Not specifically described in GVP Module V (rev 2), but based on information provided during the EMA information day/feedback from EU health authorities

Overall, EU HAs were in agreement with the removals or reclassifications of safety concerns proposed by Janssen. In some cases, EU HAs suggested modifications beyond those proposed or specifically described in GVP Module V (Rev.2). For instance, it was requested that safety concerns with a similar etiology that could be addressed through the same measures (e.g., subtypes of malignancies or infections) be grouped together as a single safety concern. In other cases, in particular for established products, EU HAs requested harmonization of safety concerns across an entire therapeutic class.

## Discussion

### Guideline on Good Pharmacovigilance Practices Module V (Revision 2)

Marketing authorization applicants, marketing authorization holders, and EU HAs have a shared interest in minimizing risks for any given medicinal product and improving the benefit-risk balance for patients within the context of risk management planning. Ideally, risk management planning should be targeted and based upon a risk-proportionate set of activities that directs resources to areas where the need for additional information and risk minimization is greatest, without placing undue burden on healthcare providers and patients. The implementation of GVP Module V (Rev 2) in 2017 provided the framework to achieve this goal. Only those safety concerns that are likely to impact the benefit-risk balance and that require active management in terms of further characterization and/or specific risk minimization should be included in the Safety Specification of the RMP.

GVP Module V (Rev 2) stands out from previous RMP guidance, which promoted lengthy lists of safety concerns that tended to increase in number over time. The RMP is no longer regarded as a “safety haven” characterized by an all-inclusive, but not actionable, list of safety concerns. Instead, the RMP is a living document that is expected to be re-evaluated and fine-tuned over the product life cycle in light of increasing product knowledge. Importantly, inclusion of any safety concern in the RMP should be a thoughtful and data-driven process.

While GVP Module V (Rev 2) represents a welcome paradigm shift in risk management planning over the product life cycle, it also poses a challenge to marketing authorization applicants, marketing authorization holders, and EU HAs alike, all of whom are tasked with applying the guidance in a rational and consistent manner.

In contrast to its predecessor, GVP Module V (Rev 2) is less didactic and allows for greater interpretation, with consistent implementation of the guidance largely left to the discretion of marketing authorization applicants and marketing authorization holders. In response to this challenge, Janssen developed a new approach, which includes the algorithms described herein, that may be applied reliably across product portfolios.

### The Janssen algorithm-driven Risk Management Plan assessment process

The algorithm-driven process developed by Janssen for the removal and reclassification of safety concerns is a structured, data-driven, and regulatory-compliant approach that promotes consistent interpretation and application of GVP Module V (Rev 2) across products regardless of product type and life-cycle stage. Each RMP is periodically re-evaluated as product knowledge is accrued and/or relevant PV activity milestones are achieved.

Upon incorporation of the EU HA assessment, application of the algorithm-driven process to 22 Janssen product RMPs resulted in a median % reduction of 82.4% (IQR 55.0%, 93.8%) in the total number of safety concerns. In the majority of cases, EU HAs accepted the proposals for the removal or reclassification of safety concerns.

Although the algorithms provide the benefit of a consistent, science-driven, and regulatory-compliant approach, they cannot address those limitations that apply to risk assessment in general. Supportive data on adverse reactions are collected from multiple sources, with varying levels of quantity and quality, complicating the evaluation of their seriousness, relatedness, and relevance. Not every adverse reaction is considered an important risk for the product in a given therapeutic context. Thus, careful deliberation and documentation of decisions are critical.

### Recommendations and insights based on the Janssen experience

Prior to applying the algorithms to an RMP, vigorous data collection (as outlined in Fig. 5) is recommended so that re-evaluation of the RMP can be accomplished as efficiently as possible and any proposed revisions may be appropriately justified. As a general rule, safety concerns with no associated activities in the PV and Risk Minimization Plans and for which there is no reasonable expectation that further investigation can provide additional characterization should be considered for removal from the RMP.

For mature products that are part of a therapeutic class with an established safety profile, EU HAs may request harmonization of safety concerns across the class. Also, risks that share the same etiology and that can be addressed through the same PV activities and/or RMMs may be grouped under a single safety concern.

In the past, marketing authorization applicants and marketing authorization holders tended to include populations excluded from the clinical development program as MI in the RMP, based solely on an absence of data. This practice contrasts with the requirements for MI as set forth in GVP Module V (Rev 2), i.e., if postauthorization use in the unstudied population is expected, the population must be included within the target indication and a different safety profile in the population must be suspected. A scientific rationale is needed for any population included as MI in the RMP (see Table 4 for examples).

Consequently, use in an unstudied population no longer automatically constitutes a safety concern. For example, use in patients with hepatic or renal impairment should be included as MI only if the product is metabolized hepatically or excreted renally and, consequently, different risks could be anticipated in these patients. Similarly, use in pregnant women should be included as MI only if women of childbearing potential are within the approved indication. Off-label product use, including use in patients for whom the product is contraindicated, is no longer considered MI. Rather, if the product is likely to be used outside the approved indication and an important risk arising from such use is anticipated, the risk should be included as a safety concern only if it is not already an IIR or IPR for the product. It should be made clear that this safety concern is associated specifically with off-label use. Any MI included in the RMP should be reassessed at PV activity milestones, as this designation may no longer apply as postmarketing experience increases and additional data become available.

In addition to evaluating safety concerns for possible removal or reclassification, it is important that product-specific RMP teams assess the relative burden versus benefit of the associated risk management activities. Understanding and assessing the burden on patients, healthcare professionals, and the wider healthcare system is an important consideration in RMP preparation and revision [7].

As GVP Module V (Rev 2) represents a markedly different approach to risk management planning compared with earlier guidance, continued learning on the part of marketing authorization applicants, marketing authorization holders, and EU HAs is to be expected. Engaging in an open dialogue, addressing potential inconsistencies, and questioning ambiguous decisions when appropriate has proven helpful in this learning process.

## Conclusions

The algorithm-driven process allows for consistent interpretation and application of the GVP Module V (Rev 2) guidance, which enables product teams to develop an actionable RMP using a thoughtful, evaluative, science-based approach that considers all available evidence. It is important that experience with GVP Module V (Rev 2) be shared among Industry stakeholders and EU HAs to move the field of risk management forward. By publishing the Janssen experience, other stakeholders may glean insights to support the development or refinement of their own RMP processes. Sharing lessons learned will ultimately strengthen the approach to risk management, to the benefit of the patients.

## Data Availability

All data analyzed and generated during this study are included in this publication.

## References

[1] European Medicines Agency. Legal framework: pharmacovigilance, https://www.ema.europa.eu/en/human-regulatory/overview/pharmacovigilance/legal-framework-pharmacovigilance (2020).

[2] European Medicines Agency and Heads of Medicines Agencies. Guideline on good pharmacovigilance practices (GVP) Module V – Risk management systems, EMA/838713/2011, 20 February 2012. https://www.ema.europa.eu/en/documents/scientific-guideline/draft-guideline-good-pharmacovigilance-practices-module-v-risk-management-systems_en.pdf.

[3] European Medicines Agency and Heads of Medicines Agencies. Guideline on good pharmacovigilance practices (GVP) Module V – Risk management systems (Rev 2), EMA/838713/2011 Rev 2*, 28 March 2017. https://www.ema.europa.eu/en/documents/scientific-guideline/guideline-good-pharmacovigilance-practices-module-v-risk-management-systems-rev-2_en.pdf.

[4] European Medicines Agency. Guidance on the format of the risk management plan (RMP) in the EU – in integrated format, EMA/164014/2018 Rev.2.0.1 accompanying GVP Module V Rev.2, 31 October 2018. https://www.ema.europa.eu/en/documents/regulatory-procedural-guideline/guidance-format-risk-management-plan-rmp-eu-integrated-format-rev-201_en.pdf.

[5] Santoro A, Genov G, Spooner A (2017). Promoting and protecting public health: how the European Union pharmacovigilance system works. Drug Saf.

[6] European Medicines Agency and Heads of Medicines Agencies. Guideline on good pharmacovigilance practices (GVP) Annex I – Definitions (Rev 4), EMA/876333/2011 Rev 4*, 9 October 2017. https://www.ema.europa.eu/en/documents/scientific-guideline/guideline-good-pharmacovigilance-practices-annex-i-definitions-rev-4_en.pdf.

[7] European Medicines Agency and Heads of Medicines Agencies. Guideline on good pharmacovigilance practices (GVP) Module XVI – Risk minimisation measures: selection of tools and effectiveness indicators (Rev 2), EMA/204715/2012 Rev 2*, 28 March 2017. https://www.ema.europa.eu/en/documents/scientific-guideline/guideline-good-pharmacovigilance-practices-module-xvi-risk-minimisation-measures-selection-tools_en-3.pdf.

[8] European Commission (2010). Directive 2010/84/EU of the European parliament and of the council of 15 December 2010 amending, as regards pharmacovigilance, directive 2001/83/EC on the community code relating to medicinal products for human use. Official J EU.

[9] European Commission (2010). Regulation (EU) No 1235/2010 of the European parliament and of the council of 15 December 2010 amending, as regards pharmacovigilance of medicinal products for human use, regulation (EC) No 726/2004 laying down community procedures for the authorisation and supervision of medicinal products for human and veterinary use and establishing a European medicines agency, and regulation (EC) No 1394/2007 on advanced therapy medicinal products. Official J EU.

[10] European Commission (2012). Commission implementing regulation (EU) No 520/2012 of 19 June 2012, on the performance of pharmacovigilance activities provided for in regulation (EC) no 726/2004 of the European parliament and the council and directive 2001/83/EC of the European parliament and the council. Official J EU.

[11] European Medicines Agency. Good pharmacovigilance practices. https://www.ema.europa.eu/en/human-regulatory/post-authorisation/pharmacovigilance/good-pharmacovigilance-practices (2020).

[12] European Medicines Agency and Heads of Medicines Agencies. Guideline on good pharmacovigilance practices (GVP) Module V – Risk management systems (Rev 1), EMA/838713/2011 Rev 1* (superseded version), 15 April 2014. https://www.ema.europa.eu/en/documents/scientific-guideline/guideline-good-pharmacovigilance-practices-module-v-risk-management-systems-rev1-superseded_en.pdf.

[13] Von Bruchhausen T and Schirp S. Medical Writing 2017;26(3):48–51. https://journal.emwa.org/observational-studies/ema-releases-the-revised-good-pharmacovigilance-practices-module-v-updated-guidance-on-risk-management-plans/.

[14] Akula A. EMA’s revised format for risk management plans – What you need to know. Pharm Online, https://www.pharmaceuticalonline.com/doc/ema-s-revised-format-for-risk-management-plans-what-you-need-to-know-0001 (17 September 2018).

[15] European Medicines Agency and Heads of Medicines Agencies. Guideline on good pharmacovigilance practices (GVP) Module VII – Periodic safety update report (Rev 1), EMA/816292/2011 Rev 1*, 9 December 2013. https://www.ema.europa.eu/en/documents/scientific-guideline/guideline-good-pharmacovigilance-practices-gvp-module-vii-periodic-safety-update-report_en.pdf.

[16] Esslinger S, Otero-Lobato M, Noël W, et al. Use of post-approval safety studies in the evaluation of safety concerns listed in the European risk management plan (EU-RMP) of two mature TNF inhibitors (TNFi). In: 35th International Conference on Pharmacoepidemiology and Therapeutic Risk Management. Philadelphia PA; 2019. p. 24–8 poster 972.

